# Highly Efficient, One Pot, Solvent and Catalyst, Free Synthesis of Novel Quinazoline Derivatives under Ultrasonic Irradiation and Their Vasorelaxant Activity Isolated Thoracic Aorta of Rat

**DOI:** 10.22037/ijpr.2019.1100675

**Published:** 2019

**Authors:** Azar Purkhosrow, Azadeh Khalili, Anthony Chih Ho, Saghar Mowlazadeh Haghighi, Shima Fakher, Ali Khalafi-Nezhad

**Affiliations:** a *Department of Pharmacology, School of Medicine, Shiraz University of Medical Sciences, Shiraz, Iran. *; b *Department of Physiology and Pharmacology, School of Medicine, Alborz University of Medical Sciences, Karaj, Iran. *; c *Evidence-based Phytotherapy and Complementary Medicine Research Center, Alborz University of Medical Sciences, Karaj, Iran.*; d *Department of Chemistry and Biochemistry, School of Science, University of Arizona, Tucson, Arizona, United States.*; e *Department of Chemistry, School of Science, Shiraz University of Science, Shiraz, Iran.*

**Keywords:** Quinazoline, Ultrasonic irradiation, Vasorelaxant activity, Thoracic aorta of rat, One pot

## Abstract

New quinazoline derivatives were prepared by one pot reaction of anthranilic acid, acetic anhydride and primary amines, under ultrasonic irradiation. As a result, Ultrasonic irradiation has led to affordable, clean synthesis of a variety of target compounds in much higher yields, than traditional methods. This method has numerous advantages: such as higher yields, shorter reactions time, and easier work-up. Several structural classes among these compounds were identified to have vasorelaxant activity. In this respect, all of the newly synthesized quinazolinone derivatives displayed vasorelaxant properties on the isolated thoracic rat aorta. The IC_50 _of compounds **2a** (-6.00 ± 0.55), **2g** (-7.31 ± 0.94), **2n** (-7.15 ± 0.81) and **2p** (-7.77 ± 0.31) was comparable to that seen in the Acetylcholine (-7.13 ± 0.14). The structures of the newly synthesized compounds were confirmed by IR, ^1^H NMR,^ 13^C NMR, mass spectral studies, elemental analysis, and melting point.

## Introduction

Quinazolinones are a class of heterocyclic compound that contain a pyrimidine nucleus in their structure (1). The Quinazolinones function in a wide range of biological properties: such as a sedative, anesthetic, anticancer, and muscle relaxant (2-6). Given such advantageous properties, the synthesis of novel quinazolinone derivatives as potent antihypertensive compounds is warrant for further in-depth studies. Aromatization ring expansion and cycloaddition reactions have been demonstrated as potential to target those multiple functions (7-8).

One of the most frequently encountered heterocycles in medicinal chemistry is 3-substituted 2-methyl quinazoline-4(3*H*)-ones. From literatures, research revealed that the presences of the substituted aromatic ring at position 3, and the methyl group at position 2, are requisites for vasorelaxant activities (9). Recently, several methods have been developed for synthesizing 3-substituted 2-methyl quinazoline-4(3*H*)-one systems (10). The most common methods for the preparation of quinazolinones involve amidation of anthranilonitrile, anthranilic acid, or anthranilamide- followed by cyclization of the resulting intermediate. The 4(3*H*)-Quinazolines were prepared using silica sulfuric acid, PCl_3_, and Zn/HCOONH_4_ under microwave irradiation (11-13). Most of these multi-step procedures have significant drawbacks: such as longer reaction times lower product yields, harsher reaction conditions, and the usage of expensive and environmentally toxic catalysts or reagents. The development of a simplistic and efficient method for the synthesis of 3-substituted 2-methyl quinazoline-4(3*H*)-ones is therefore desirable.

This study was focused on the synthesis of novel quinazolinone derivatives and the study of vasorelaxant effects of 3-substituted 2-methyl quinazoline-4(3*H*)-ones. The synthesis of these compounds was performed by the reactions of anthranilic acid and acetic anhydride, with selected aromatic/aliphatic primary amines under Ultrasonic irradiation in one pot. The yield of this reaction ranged from fair, to excellent. Due to the fact that ultrasound irradiation is able to activate numerous organic reactions, ultrasonic chemistry has received increased recognition in the recent years (14). In addition, ultrasound irradiation benefits to an acceleration of organic reactions in homogeneous and heterogeneous systems than that of conventional methods (15–24). The ultrasonic sonochemical phenomenon originates from the interaction between a suitable field of sound waves and a potentially reacting chemical system (25-27). 

## Experimental

*Chemistry*

All chemicals used in the present study were of analytical grade, purchased from Sigma, Aldrich and Merck chemical Co. All the solvents were used after distillation, and dried according to their standard methods. All reactions were monitored by thin laer chromatography (TLC) on precoated silica gel Poly Gram SILG/UV254 plates; the spots were visualized with UV light. Sonication was performed using a Dr. Hielscher UP200Hultrasonic instrument with a frequency of 24 kHz and nominal power of 600 W/cm2. Melting points were measured in open capillaries tubes in a Barnstead Electrothermal 9100 BZ circulating oil melting point apparatus. Elemental analyses were performed on a thermos sfinnigan flash EA1112-1CHNS. IR spectra were recorded on a Perkin–Elmer FT-IR 240-C spectrophotometer using KBr optics. Mass spectra were recorded on a FINNIGAN-MAT 8430 mass spectrometer operating at 70 eV.^ 13^C nuclear magnetic resonance (^13^C NMR) and ^1^H nuclear magnetic resonance (^1^H NMR) spectra were recorded for the compounds on Advance bruker (250 MHz) instrument. Chemical shifts are reported in parts per million (ppm) using tetramethylsilane (TMS) as an internal standard.

*General experimental procedure for the preparation of 3-substituted 2-methyl quinazoline-4(3H)-ones*

The mixture of anthranilic acid (1 mmol), acetic anhydride (1.2 mmol) with selected aromatic/aliphatic amines (1 mmol) were reacted under ultrasonic irradiation. The reactions were performed in a water bath. After completion of reaction (monitored by TLC), the reaction mixture was washed with Chloroform (3 × 10 mL). The Chloroform was evaporated to give the crude product, which was recrystallized from ethanol. All the products obtained were fully characterized by spectroscopic methods such as IR, ^1^H NMR, ^13^C NMR, elemental analysis, and mass spectroscopy and have been identified by the comparison of the reported spectral data. 

*The spectral data for synthesized compounds*

*2-methyl-3-phenylquinazoline-4(3H)-one (* ***2a*** *)*

White solid, mp 145 °C. IR (KBr, cm^-1^): 3100 (s) , 2921 (s), 1675 (s), 1582 (s), 755 (s). ^1^H NMR (250 MHz, DMSO-d_6_) δ (ppm): 2.09 (s, 3H), 7.39-7.64 (m, 8H), 8.05 (d, J = 7.5 Hz, 1H).^13^C NMR (62.9 MHz, DMSO-d_6_) δ (ppm): 24.0 , 120.4, 124.5, 126.2, 126.3, 126.6, 128.4, 128.9, 129.5, 137.8, 147.2, 154.3, 161.4. MS (m/z): 236 (M^+^), 149 (21.9%), 97 (22.3%), 77 (67.2%), 57 (base peak, 100%). Anal. Calcd for C_15_H_12_N_2_O (236.27): C,76.25; H,5.12; N,11.86. Found: C, 79.81; H, 5.03; N, 11.75.

*2-methyl-3-(4-methylpiperazin-1-yl)quinazolin-4(3H)-one (* ***2b*** *)*

 Yellow solid, mp 79.8 °C. IR (KBr, cm^-1^): 3100 (s), 3001 (w), 2931 (s), 1674 (s), 1589 (s), 1458 (m), 779 (s). ^1^H NMR (250 MHz, DMSO-d_6_) δ (ppm): 1.98 (s, 3H), 2.53 (s, 3H), 2.76-2.81 (m, 8H), 7.3-7.6 (m, 3H), 8.09 (d, J = 1 Hz, 1H).^13^C NMR (62.9 MHz, CDCl_3_) δ (ppm): 22.6, 45.1, 49.6, 54.9, 120.9, 122.4, 126.2, 126.3, 134.3, 146.6, 157.6, 161.8. MS (m/z): 258 (M^+^), 236 (7.4%), 201 (4.6%), 185 (3.1%), 166 (4.8%), 137 (7.2%), 99 (18.4%), 83 (55.7%), 57 (base peak, 100%). Anal. Calcd for C_14_H_18_N_4_O (258.32): C, 65.09; H, 7.02; N, 21.69. Found: C, 65.01; H, 6.94; N, 21.61.

*3-(3-hydroxypyridin-2-yl)-2-methylquinazolin-4(3H)-one (* ***2c*** *)*

Orange solid, mp 183.8 °C. IR (KBr, cm^-1^): 3550 (m), 3100 (s), 2950 (s), 1680 (s), 1620 (s), 760 (s). ^1^H NMR (250 MHz, DMSO-d_6_) δ (ppm): 2.17 (s, 3H), 7.29 (t, J = 7.5 Hz, 1H), 7.4 (d, J = 7.5 Hz, 1H), 7.62-7.75 (m, 3H), 8.25 (d, J = 1.5 Hz, 1H), 8.68 (d, J = 10.5 Hz, 1H), 10.8 (s, 1H). ^13^C NMR (62.9 MHz, DMSO-d_6_) δ (ppm): 24.9, 116.3, 119.8, 122.4, 126.0, 128.4, 131.0, 133.9, 140.8, 141.5, 147.8, 151.0, 152.5, 161.5. MS (m/z): 253 (M^+^), 192 (1.5%), 161 (1.7%), 95 (12.9%), 69 (base peak, 100%). Anal. Calcd for C_14_H_11_N_3_O_2_ (253.26 ): C, 66.40; H, 4.38; N, 16.59. Found: C, 66.31; H, 4.30; N, 16.54.

*N,2-dimethyl-4-oxoquinazoline-3(4H)-carboxamide (* ***2d*** *)*

Yellow solid, mp 181.2 °C. IR (KBr, cm^-1^): 3400 (m), 3102 (s), 3000 (s), 1645 (s), 1596 (s), 775 (s). ^1^H NMR (250 MHz, DMSO-d_6_) δ (ppm): 2.1 (s, 3H), 3.45 (s, 3H), 7.49-7.71 (m, 3H), 8.42 (d, J = 7.5 Hz, 1H), 11.05 (s, 1H).^13^C NMR (62.9 MHz, DMSO-d_6_) δ (ppm): 21.3, 24.9, 122.4, 126.0, 126.3, 130.9, 134.1, 148.7, 161.7, 168.4, 169.4. MS (m/z): 217 (M^+^), 179 (11.8%), 160 (76.3%), 137 (50.3%), 119 (base peak, 100%), 92 (54%), 69 (76.9%). Anal. Calcd for C_11_H_11_N_3_O_2_ (217.22): C, 60.82; H, 5.10; N, 19.34. Found: C, 60.74; H, 5.01; N, 19.28.

*2-(2-methyl-4-oxoquinazolin-3(4H)-yl)anthracene-9,10-dione (* ***2e*** *)*

Orange solid, mp 236.4 °C. IR (KBr, cm^-1^): 3149 (s), 2950 (s), 1705 (s), 1674 (s), 1589 (s), 779 (s). ^1^H NMR (250 MHz, DMSO-d_6_) δ (ppm): 1.7 (s, 3H), 6.9 (t, J = 7.5 Hz, 1H), 7.23 (d, J = 7.5 Hz, 1H), 7.85-8.15 (m, 8H), 8.42 (s, 1H). ^13^C NMR (62.9 MHz, DMSO-d_6_) δ (ppm): 21.1, 115.5, 120.7, 123.0, 126.5, 126.9, 127.0, 130.5, 131.0, 131.1, 135.2, 138.3, 146.5, 156.1, 159.2, 170.1, 176.5, 180.2. MS (m/z): 366 (M^+^), 223 (13.1%), 207 (12.3%), 151 (42.8%), 111 (17.4%), 83 (52.2%), 57 (base peak, 100%). Anal. Calcd for C_23_H_14_N_2_O_3_ (366.37): C, 75.40; H, 3.85; N, 7.65. Found: C, 75.31; H, 3.77; N, 7.58.

*3-benzyl-2-methylquinazolin-4(3H)-one (* ***2f*** *)*

Yellow oil. IR (KBr, cm^-1^): 3050 (s), 2860 (s), 1640 (s), 1600 (s), 1470 (m), 725 (s). ^1^H NMR (250 MHz, DMSO-d_6_) δ (ppm): 2.41 (s, 3H), 5.24 (s, 2H), 7.07-7.63 (m, 8H), 8.1 (d, J = 0.7 Hz, 1H). ^13^C NMR (62.9 MHz, CDCl_3_) δ (ppm): 23.3, 47.0, 120.3, 122.4, 127.0, 127.1, 128.6, 128.9, 134.4, 139.6, 147.3, 154.6, 162.3. MS (m/z): 250 (M^+^), 144 (22.9%), 91 (base peak, 100%), 65 (26.5%). Anal. Calcd for C_16_H_14_N_2_O (250.29): C, 76.78; H, 5.64; N, 11.19. Found: C, 76.71; H, 5.61; N, 11.12. 

*3-(4-nitrophenylamino)-2-methylquinazolin-4(3H)-one (* ***2g*** *)*

Orange solid, mp 101.1 °C. IR (KBr, cm^-1^): 3506 (w), 3190 (s), 2968 (s), 1675 (s), 1596 (s), 1502 (s), 1350 (s), 800 (s). ^1^H NMR (250 MHz, DMSO-d_6_) δ (ppm): 2.45 (s, 3H), 6.8 (d, J = 7.5 Hz, 1H), 7.49 (t, J = 3.3 Hz, 1H), 7.69 (d, J = 7.5 Hz, 1H), 7.86 (t, J = 1.16 Hz, 1H), 8.1 (d, J = 2.5 Hz, 1H). ^13^C NMR (62.9 MHz, DMSO-d_6_) δ (ppm): 21.1 , 111.7, 120.9, 125.9, 126.3, 126.6, 126.9, 134.9, 139.8, 146.5, 152.6, 156.8, 159.9. MS (m/z): 296 (M^+^), 160 (21.2%), 123 (10.1%), 97 (16.1%), 69 (base peak, 100%); Anal. Calcd for C_15_H_12_N_4_O_3_ (296.28): C, 60.81; H, 4.08; N, 18.91. Found: C, 60.75; H, 4.02; N, 18.86. 

*N-(2-methyl-4-oxoquinazolin-3(4H)-yl)benzamide (* ***2h*** *)*

White solid, mp 187.0 °C. IR (KBr, cm^-1^): 3450 (m), 3140 (s), 2950 (s), 1665 (s), 1597 (s), 795 (s). ^1^H NMR (250 MHz, DMSO-d_6_) δ (ppm): 2.36 (s, 3H), 7.5-8.1 (m, 9H), 11.63 (s, 1H). ^13^C NMR (62.9 MHz, DMSO-d_6_) δ (ppm): 21.0, 120.5, 126.4, 126.8, 126.9, 127.7, 128.8, 131.1, 132.8, 135.0, 146.5, 156.1, 159.0, 165.9. MS (m/z): 279 (M^+^), 237 (4.9%), 174 (32%), 159 (6.7%), 120 (12.3%), 77 (base peak, 100%). Anal. Calcd for C_16_H_13_N_3_O_2_ (279.29): C, 68.81; H, 4.69; N, 15.05. Found: C, 68.76; H, 4.63; N, 15.01.

*2-methyl-4-oxo-N-phenylquinazoline-3(4H)-carboxamide (* ***2i*** *)*

White solid, mp 176.0 °C. IR (KBr, cm^-1^): 3400 (m), 3130 (s), 2960 (s), 1670 (s), 1595 (s), 790 (s). ^1^H NMR (250 MHz, DMSO-d_6_) δ (ppm): 2.46 (s, 3H), 6.94 (q, J = 17.5 Hz, 1H), 7.22-7.45 (m, 8H), 8.63 (s, 1H). ^13^C NMR (62.9 MHz, DMSO-d_6_) δ (ppm): 25.1, 118.1, 121.7, 124.4, 127.4, 128.7, 129.0, 134.5, 139.6, 148.0, 152.5, 166.3, 169.1. MS (m/z): 279 (M^+^), 261 (2.2%), 213 (2.4%), 194 (2.8%), 149 (10%), 111 (18.3%), 83 (37.3%), 57 (base peak, 100%). Anal. Calcd for C_16_H_13_N_3_O_2_ (279.29): C, 68.81; H, 4.69; N, 15.05. Found: C, 68.77; H, 4.61; N, 15.00.

*2-methyl-3-(7H-purin-6-yl)quinazolin-4(3H)-one (* ***2j*** *)*

White solid, mp 154.0 °C. IR (KBr, cm^-1^): 3274 (m), 3116 (s), 2930 (s), 1689 (s), 1582 (s), 1530 (m), 750 (s). ^1^H NMR (250 MHz, DMSO-d_6_) δ (ppm): 2.1 (s, 3H), 7.09 (t, J = 4.2 Hz, 1H), 7.5 (t, J = 6.0 Hz, 1H), 7.92 (d, J = 10.0 Hz, 1H), 8.14 (s, 1H), 8.42 (d, J = 7.5 Hz, 1H), 9 (s, 1H), 11.07 (s, 1H).^13^C NMR (62.9 MHz, DMSO-d_6_) δ (ppm): 24.9, 119.8, 122.4, 126.2, 129.5, 131.5, 133.9, 134.5, 140.8, 151.2, 152.2, 157.0, 165.7, 168.4.MS (m/z): 278 (M^+^), 236 (4.4%), 197 (3.8%), 177 (6.6%), 135 (18.9%), 97 (38.6%), 81 (39.8%), 57 (base peak, 100%). Anal. Calcd for C_14_H_10_N_6_O (278.27): C, 60.43; H, 3.62; N, 30.20. Found: C, 60.40; H, 3.59; N, 30.16. 

*3-(3-hydroxyphenyl)-2-methylquinazolin-4(3H)-one (* ***2k*** *)*

White solid, mp 114.5 °C. IR (KBr, cm^-1^): 3610 (m), 3100 (s), 2900 (s), 1645 (s), 1597 (s), 720 (s). ^1^H NMR (250 MHz, DMSO-d_6_) δ (ppm): 2.13 (s, 3H), 6.7 (d, J = 2.5 Hz, 1H), 6.9 (q, J = 7.5 Hz, 1H), 7.1 (s, 1H), 7.3 (t, J = 2.5 Hz, 1H), 7.4 (t, J = 2.5 Hz, 1H), 7.6 (d, J = 7.5 Hz, 1H), 7.8 (d, J = 2.5 Hz, 1H), 8 (d, J = 7.5 Hz, 1H), 9.8 (s, 1H). ^13^C NMR (62.9 MHz, DMSO-d_6_) δ (ppm): 23.7, 115.3, 115.9, 118.7, 120.4, 126.2, 126.3, 126.5, 130.2, 134.4, 138.7, 147.2, 154.4, 158.2, 161.1. MS (m/z): 252 (M^+^), 210 (4.8%), 177 (5.9%), 139 (8.1%), 97 (39%), 58 (base peak, 100%). Anal. Calcd for C_15_H_12_N_2_O_2_ (252.27): C, 71.42; H, 4.79; N, 11.10. Found: C, 71.37; H, 4.72; N, 11.02.

*3-(6,7-dihydro-6-oxo-1H-purin-2-yl)-2-methylquinazolin-4(3H)-one (* ***2l*** *)*

White solid, mp 180.7 °C. IR (KBr, cm^-1^): 3317 (m), 3109 (s), 2900 (s), 1697 (s), 1620 (m), 1550 (m), 779 (s). ^1^H NMR (250 MHz, DMSO-d_6_) δ (ppm): 2.1 (s, 3H), 7.1 (t, J = 7.5 Hz, 1H), 7.5 (t, J = 7.5 Hz, 1H), 7.9 (d, J = 7.5 Hz, 1H), 8.4 (d, J = 7.5 Hz, 1H), 9.2 (s, 1H), 11 (s, 1H), 11.8 (s, 1H). ^13^C NMR (62.9 MHz, DMSO-d_6_) δ (ppm): 24.9, 116.4, 119.9, 122.5, 126.2, 129.6, 130.1, 131.0, 131.6, 133.9, 134.7, 140.8, 168.4, 169.4. MS (m/z): 294 (M^+^), 252 (5.3%), 213 (6.3%), 193 (7%), 96 (42%), 62 (base peak, 100%). Anal. Calcd for C_14_H_10_N_6_O_2_ (294.27): C, 57.14; H, 3.43; N, 28.56. Found: C, 57.07; H, 3.40; N, 28.51. 

*3-(4-bromophenyl)-2-methylquinazolin-4(3H)-one (* ***2m*** *)*

White solid, mp 157.2 °C. IR (KBr, cm^-1^): 310.7 (s), 2911 (s), 1665 (s), 1560 (s), 1061 (s), 741 (s). ^1^H NMR (250 MHz, DMSO-d_6_) δ (ppm): 2.00 (s, 3H), 7.39-7.66 (m, 7H), 7.72 (d, J = 10.0 Hz, 1H). ^13^C NMR (62.9 MHz, DMSO-d_6_) δ (ppm): 23.9, 120.4, 124.5, 126.2, 126.3, 126.6, 128.4, 128.9, 129.5, 137.8, 147.2, 154.3, 161.3. MS (m/z): 315 (M^+ ^+1), 236 (0.9%), 215 (22.1%), 171 (base peak, 100%), 92 (63.8%), 65 (64.6%). Anal. Calcd for C_15_H_11_BrN_2_O (315.16): C, 57.16; H, 3.52; N, 8.89. Found: C, 57.11; H, 3.44; N, 8.79.

*3-(2,4-dinitrophenylamino)-2-methylquinazoline-4(3H)-one (* ***2n*** *)*

Yellow solid, mp 181.0 °C. IR (KBr, cm^-1^): 3490 (m), 3100 (s), 3000 (s), 1675 (s), 1610 (s), 1526 (s), 1345 (s), 773 (m). ^1^H NMR (250 MHz, DMSO-d_6_) δ (ppm): 2.40 (s, 3H), 7.10 (d, J = 10.0 Hz, 1H), 7.54-8.08 (m, 3H), 8.20 (d, J = 2.5 Hz, 1H), 8.25 (d, J = 2.5 Hz, 1H), 8.92 (d, J = 5.0 Hz, 1H), 11.04 (s, 1H).^13^C NMR (62.9 MHz, DMSO-d_6_) δ (ppm): 21.1, 115.5, 120.7, 123.0, 126.5, 126.9, 127.0, 130.5, 131.0, 131.1, 135.2, 138.3, 146.5, 156.1, 159.2. MS (m/z): 341 (M^+^), 179 (11.4%), 149 (7%), 119 (64.8%), 92 (31%), 69 (base peak, 100%). Anal. Calcd for C_15_H_11_N_5_O_5_ (341.28): C, 52.79; H, 3.25; N, 20.52. Found: C, 52.71; H, 3.20; N, 20.47. 

*2-methyl-3-(phenylsulfonyl)quinazolin-4(3H)-one (* ***2o*** *)*

Yellow solid, mp 223.0 °C. IR (KBr, cm^-1^): 3110 (s), 2950 (s), 1660 (s), 1600 (s), 1320 (s), 1150 (s), 800 (s). ^1^H NMR (250 MHz, DMSO-d_6_) δ (ppm): 2.10 (s, 3H), 7.1 (q, J = 7.0 Hz, 1H), 7.51-7.58 (m, 5H), 7.92-7.96 (m, 3H).^13^C NMR (62.9 MHz, DMSO-d_6_) δ (ppm): 24.9, 116.4, 119.9, 122.5, 127.3, 127.4, 131.0, 132.1, 133.9, 140.7, 148.0, 168.4, 169.9. MS (m/z): 300 (M^+^), 237 (18%), 159 (7.3%), 141 (6.4%), 59 (base peak, 100%). Anal. Calcd for C_15_H_12_N_2_O_3_S(300.33): C, 59.99; H, 4.03; N, 9.33. Found: C, 59.91; H, 4.01; N, 9.27. 

*3-(diphenylmethyleneamino)-2-methylquinazolin-4(3H)-one (* ***2p*** *)*

Yellow solid, mp 131.6 °C. IR (KBr, cm^-1^): 3050 (s), 2931 (s), 1666 (s), 1566 (s), 771 (s). ^1^H NMR (250 MHz, DMSO-d_6_) δ (ppm): 2.21 (s, 3H), 7.26-7.55 (m, 9H), 7.78-7.82 (m, 4H), 8 (d, J = 5 Hz, 1H). ^13^C NMR (62.9 MHz, DMSO-d_6_) δ (ppm): 22.3, 120.2, 126.2, 127.4, 128.7, 129.6, 129.8, 130.0, 131.6, 133.9, 147.0, 159.2, 159.0, 165.2. MS (m/z): 339 (M^+^), 311 (5.2%), 297 (6.4%), 180 (7.5%), 166 (11.3%) 159 (14.8%), 76 (base peak, 100%). Anal. Calcd for C_22_H_17_N_3_O(339.39): C, 77.86; H, 5.05; N, 12.38. Found: C, 77.81; H, 5.01; N, 12.30.

*2-methyl-3-(1H-1,2 ,4-triazol-3-yl)quinazolin-4(3H)-one (* ***2q*** *)*

White solid, mp 166.5 °C. IR (KBr, cm^-1^): 3494 (m), 3050 (s), 2900 (s), 1689 (s), 1596 (s), 750 (s). ^1^H NMR (250 MHz, DMSO-d_6_) δ (ppm): 2.1 (s, 3H), 7.1 (t, J = 7.5 Hz, 1H), 7.5 (t, J = 7.5 Hz, 1H) 7.9 (d, J = 7.5 Hz, 1H), 8.4 (d, J = 10.0 Hz, 1H), 8.7 (s, 1H), 11.12 (s, 1H). ^13^C NMR (62.9 MHz, DMSO-d_6_) δ (ppm): 25.4, 115.0, 116.7, 120.2, 123.4, 131.6, 134.1, 141.3, 151.9, 153.9, 161.8. MS (m/z): 227 (M^+^), 199 (10.4%), 159 (7%), 117 (44.5%), 92 (31%), 69 (base peak, 100%). Anal. Calcd for C_11_H_9_N_5_O(227.22): C, 58.14; H, 3.99; N, 30.82. Found: C, 58.14; H, 3.99; N, 30.82. 

*Pharmacological study*

*Vasorelaxant activity*

The vasorelaxant activity of the synthesized quinazolinones was evaluated on isolated thoracic aorta of rats. In the present study, to evaluate the relaxation and contraction response we use Acetylcholine (Ach) and Phenylephrine (PE) as standard compounds, respectively. The effects of our new compounds on vascular function have been compared to that of Ach and PE. Direct Vasorelaxant activity of new quinazoline compounds (2a-2q) were analyzed on isolated thoracic aorta of rats.

Using intraperitoneal injection of Ketamine (60 mg/kg) and Xylazine (8 mg/kg), the male Sparque-Dawley rats (200-250 g) were anaesthetized. Afterwards, the thoracic aorta was cleansed of surrounding fat and connective tissues; cut into four rings of approximately 2-3 mm length, which was mounted on hooks connected to force transducer in isolated tissue organ bath (K30, Hugo Sachs Electronik, Germany) filled with 20 mL physiological solution of the following composition (mmol/L): NaCl 118, KCl 4.7, KH_2_PO_4_ 1.2, CaCl_2_ 2.5, MgSO_4_ 1.2, NaHCO_3_ 25, D-glucose 11.1 kept at 37 °C. and bubbled constantly with 95% O_2_ and 5% CO_2_ (pH 7.4). Tension was recorded by a four channel polygraph (model 705/1, Hugo Sachs Electronik, Germany). The tissues were allowed to stabilize for 60 min, during which they were washed every 20 min. Afterwards, ring was precontracted using the Phenylephrine (PE: 10^-6^ M). Dose-relaxation response to new compounds (10^-9^-10^-4^ M) was performed at the plateau of contractile response to PE. Acetylcholine was used as a reference standard for vasodilating activity. The comparison of the groups was performed using IC_50_ and maximum response (E_max_). 

## Results and Discussion

**Figure 1:**
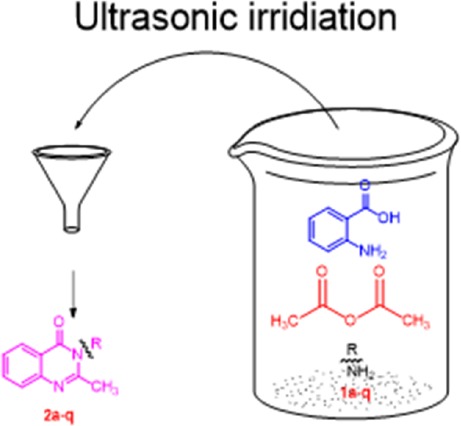
The synthesis of ouinazolione compounds by ultrasonic irridiation

To our knowledge, all quinazolinone derivatives that were prepared in this study are novel compounds. They were developed in one pot reactions, under ultrasonic irradiation. The products were synthesized from commercially available materials. Ultrasonic irradiation has been widely applied in organic reactions. Replaced the traditional stirring method, ultrasonic irradiation accelerated a variety of synthetic transformations with a time- and energy-saving experimental design. As ultrasound generates intense turbulence and micro-scale liquid circulation currents, it results in a homogenous mixture at micro level. Considering the advantages of ultrasonic irradiation, we have designed an efficient and practical procedure for the synthesis of 3-substituted 2-methyl quinazoline-4(3*H*)-ones with anthranilic acid, acetic anhydride, and primary amines under ultrasonic irradiation (Figure 1).

**Figure 2:**
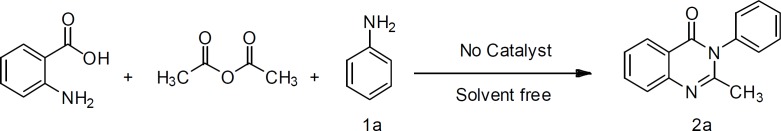
Reaction model for the synthesis of the novel quinazolinone compounds

**Figure 3:**
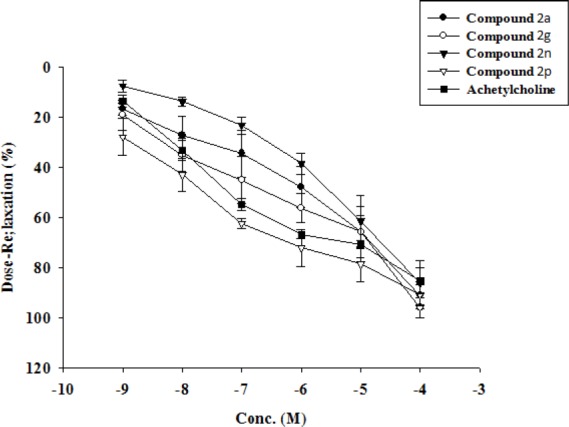
Ach relaxation response curves (n = 7 in each group) in isolated aortic rings from sparaq dawley rats for 4 new quinazoline compounds (**2a**, **2g**, **2n** and **2p**). The response (relaxation%) was calculated as the percentage of maximal responses to Ach in the control group

**Table 1:** Study of effect of different catalysts on the model reaction in refluxed solvents

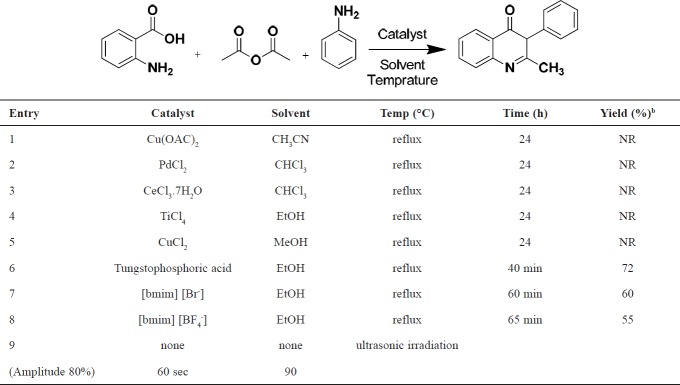

**Table 2:** Under ultrasonic irradiation and solvent free

**Yield (%)** a	**Time (min)**	**Amplitude (%)**	**Cycle**	**Entry**
40	4	40	1	1
73	2	60	1	2
90	1	80	1	3

a Isolated yield.

**Table 3:** Results of three-component condensation reaction

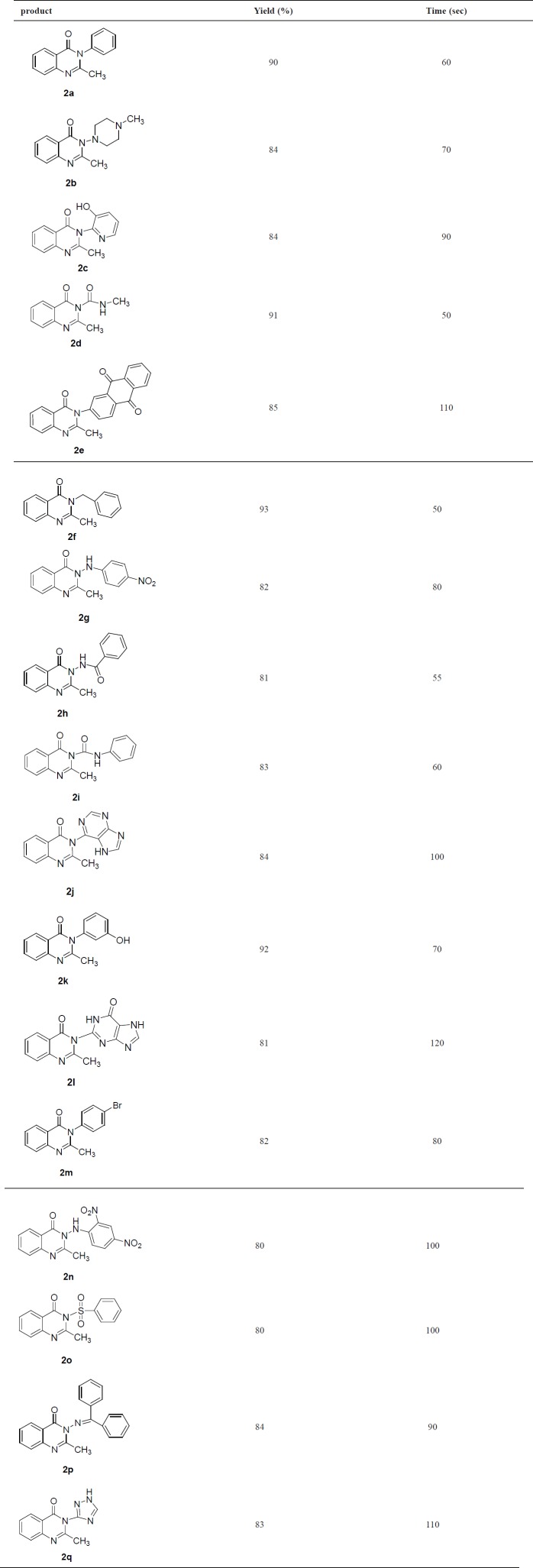

Products of three-component condensation reaction between anthranilic acid, acetic anhydride and primary amines under Ultrasonic Irridiation. Reaction conditions: anthranilic acid (1 mmol), acetic anhydride (1.2 mmol) and amine (1 mmol). All yields are isolated yield.

**Table 4:** Comparison between vasorelaxant active synthesized quinazolinone and acetylcholine chloride on isolated thoracic rat aorta

**No. of compound**	**IC** **50**	**E** **max**
**2a**	-6.00 ± 0.55	91.1 ± 5.5
**2b**	-5.50 ± 0.47*	95.3 ± 2.0
**2c**	-4.54 ± 0.67*	84.8 ± 8.3
**2d**	-5.33 ± 0.24*	77.7 ± 7.5
**2e**	-5.65 ± 0.59*	57.8 ± 9.9*
**2f**	-5.30 ± 0.50*	81.8 ± 6.7
**2g**	-7.31 ± 0.94	86.4 ± 4.0
**2h**	-3.90 ± 0.28*	50.0 ± 4.5*
**2i**	-5.03 ± 0.76*	76.6 ± 10
**2j**	-5.56 ± 0.40*	77.6 ± 13
**2k**	-5.24 ± 0.24*	86.1 ± 9.0
**2l**	-5.38 ± 0.72*	78.0 ± 4.9
**2m**	-5.45 ± 0.21*	84.1 ± 8.3
**2n**	-7.15 ± 0.81	86.1 ± 8.9
**2o**	-5.24 ± 0.47*	60.5 ± 4.4*
**2p**	-7.77 ± 0.31	90.7 ± 3.9
**2q**	-5.16 ± 0.60*	65.0 ± 9.1
**Ach**	-7.13 ± 0.14	85.31 ± 5.3

* Denotes significantly different compared to Ach.

We have examined the synthesis of 2-methyl-3-phenylquinazoline-4(3*H*)-one (**2a**) from Anthranilic acid (1 mmol) , acetic anhydride (1.2 mmol) and aniline (1 mmol) as a model reaction. The product (**2a**) was synthesized in solvent free condition under ultrasonic irradiation (Figure 2).

In our initial study, the reaction was incorporated with Cu(OAC)_2_ in CH_3_CN solvent, where **2a** did not lead to any products even after 24 h (Table 1, entry 1). The reaction was also transferred in CHCl_3_ using PdCl_2_, CeCl_3, _7H_2_O as the catalyst, but no reactivity differences were observed (Table 1, entries 2 and 3). Similar results were also obtained with TiCl_4_ or CuCl_2 _as the catalyst in protic solvent at reflux condition (Table 1, entries 4 and 5). The yield of product was enhanced when an ionic liquid, such as ethanol, was used as catalyst at reflux condition (Table 1, entries 7 and 8). As it is shown in this table, under refluxing conditions, higher reaction time is required. We have tested the reaction in different types of solvent, and the results were shown that in solvent free conditions, favorable yield was obtained in comparison with the other solvents (Table 1, entry 9). However, in spite of their potential utility, some of the reported methods involved the use of Lewis acids (such as TiCl_4_, entry 4). These traditional acids are corrosive, and produce significant amounts of waste; which limits their usefulness and causes serious environmental and safety concerns. Furthermore, with longer reaction times, lower yields, and toxic organic solvents, are disadvantages of some of these mentioned methods (such as TiCl_4_). Due to their importance and useful properties, the development of an efficient, environmentally benign method for the preparation of these widely used heterocyclic compounds is a major challenge in synthetic organic chemistry. Consequently, a method that uses TiO_2_ as the catalyst should greatly contribute to the development of an environmental friendly process. Based on research, there are no reports on the use of TiO_2_ as a heterogeneous catalyst ultrasonic irradiation for the synthesis of substituted quinolones [28-30]. This is the first of its kind. Finally, the reaction proceeded under solvent-free conditions and ultrasound irradiation (Amplitude 80%); interestingly, it was completed at 60 sec and produced the desired product in 90% yield (Table 2).

For these reactions, the ultrasound reactor was set at 80% amplitude, with a pulse cycle of 1, frequency set to 24 kHz, and with an output power of 600 W. The results from Table 2 showcase the effectivness of ultrasonic irridiation on the development of this reaction. With its ability to distribute sound evenly throughout the bath, there is no need of other technology to operate the bath, and it works well for high frequency applications. Our results dsiplayed an increase in the yield complemented by an increase of percentage of Amplitude (80%).

Various 3-substituted 2-methyl quinazoline-4(3*H*)-one derivative were synthesized under ultrasonic condition with a green procedure in excellent yields. Ultrasonic irradiation conveniently developed the one pot condensation reaction in solvent-free conditions. The results are summarized in Table 3. As it is shown in this table (Table 3), both aliphatic and aromatic amines led to desired 4(3*H*)-quinazolinone under ultrasonic irradiation. These reactions also undergo an intramolecular nucleophilic addition across the primary amines group- leading to formation of the quinazolinone ring in excellent yields. Due to the nucleophilicity of the amines with electron- withdrawing groups decrease, so the yields of primary amines with electron-donating groups are superior to those of electron-withdrawing groups (Table 3). Thin layer chromatography (TLC) was run throughout the reaction to optimize the reaction for purity and completion. The structure of the synthesized compounds is determined on the bases of spectroscopic data, including, IR, NMR, MS, elemental analysis and melting point as reported in experimental section. The findings of our study revealed that all of the newly synthesized quinazoline derivatives exhibited vasorelaxant activity when tested on the isolated thoracic rat aorta. These compounds have the same basic structure with prazosin that acts as antihypertensive agents due to *α*_1_-antagonist activity. It seems that the substitution of R groups with ones that are seen in compounds **2a**, **7g**, **2n** and **2p** improves their biological activity compared to the other groups. Among these four compounds, phenyl group is prevalent. As a conclusion, it seems that the presence of phenyl group on that situation is a key characteristic for the improvement of vasorelaxant activity among compound **2p** with two phenyl groups, resulting in having a higher, but not a significant vasorelaxant effect. In addition, the high lipophilicity and ability to penetrate the thoracic aorta tissue could be helpful in decreasing the dose of agents using as antihypertensive drugs. On the other hand, the groups consisting of a NO_2_ on that phenyl substitution have slightly better vasodilating activity. Although Prazosin is the parent compound, it might be involved in other vasodilating pathways. The presence of the nitro receptors on vascular smooth muscle has been postulated by Needleman and Johnson [27]. These investigators suggested that the nitro – receptor interaction is accompanied by the oxidation of critical receptor sulfhydryl groups, which initiate vascular relaxation (Entries **2g**, **2n**). Consequently, substituting Aniline derivatives as a substrate are necessary for synthesis of varieties quinazolines. Using Aniline as its derivatives provides researchers with a cheap, convenient, and easy to hand substitute. We have selected different derivatives of Aniline with electron donating and withdrawing groups to make potential vasorelaxant quinazolines. They are incredibly efficient on biological activity of compounds and they improve Vasorelaxant activity of quinazoline analougues (**2a**, **2g**, **2n** and **2p**). The outcomes show that the IC_50 _of mentioned compounds are commensurate with Ach in vasorelaxant activity.

*Pharmacological study: Findings*

Table 4 shows comparison of the vasorelaxant activity of new quinazoline compounds on the isolated thoracic rat aorta. The IC_50_ of compounds **2a**, **2g**, **2n,** and **2p** was comparable to that of seen in Ach (-7.13 ± 0.14) but the other value was significantly lower compared to Ach IC_50_. Most of the new compounds (except: **2c**, **2h** and **2i**) did have comparable efficacy or maximal response to that of Ach (85.31 ± 5.32). These results indicate that despite the shift to the right translocation of the dose-response curve of quinolones compared to Ach, the relaxation efficacy is as great as for Ach (Figure 3).

## Conclusion

In conclusion, we have established a novel efficient and facile method for the synthesis of quinazoline derivatives at ambient conditions, under ultrasonic waves. Ultrasound induces a notable acceleration for reactions, resulting in a significant decrease in reaction times. In addition, the synthesis of a quinazoline reaction, with high yields is achieved. Ultrasonic irradiation efficiently promoted one-pot condensation reaction between anthranilic acid, acetic, anhydride, and amine derivatives in n solvent and catalyst free conditions. The findings of our study revealed that all of the newly synthesized quinazoline derivatives displayed vasorelaxant activity on the isolated thoracic rat aorta.
